# Human β-Defensin 2 and Its Postulated Role in Modulation of the Immune Response

**DOI:** 10.3390/cells10112991

**Published:** 2021-11-03

**Authors:** Martyna Cieślik, Natalia Bagińska, Andrzej Górski, Ewa Jończyk-Matysiak

**Affiliations:** 1 Bacteriophage Laboratory, Hirszfeld Institute of Immunology and Experimental Therapy, Polish Academy of Sciences, 53-114 Wrocław, Poland; martyna.cieslik@hirszfeld.pl (M.C.); natalia.baginska@hirszfeld.pl (N.B.); ewa.jonczyk-matysiak@hirszfeld.pl (E.J.-M.); 2 Phage Therapy Unit, Hirszfeld Institute of Immunology and Experimental Therapy, Polish Academy of Sciences, 53-114 Wrocław, Poland; 3 Infant Jesus Hospital, The Medical University of Warsaw, 02-006 Warsaw, Poland

**Keywords:** human β-defensin, hBD-2, small antimicrobial peptides, innate immunity, epithelium

## Abstract

Studies described so far suggest that human β-defensin 2 is an important protein of innate immune response which provides protection for the human organism against invading pathogens of bacterial, viral, fungal, as well as parasitical origin. Its pivotal role in enhancing immunity was proved in infants. It may also be considered a marker of inflammation. Its therapeutic administration has been suggested for maintenance of the balance of systemic homeostasis based on the appropriate composition of the microbiota. It has been suggested that it may be an important therapeutic tool for modulating the response of the immune system in many inflammatory diseases, offering new treatment modalities. For this reason, its properties and role in the human body discussed in this review should be studied in more detail.

## 1. Introduction

One of the most important components of innate immunity is the ability to synthetize and release small antimicrobial peptides, with proven activity against different pathogens, such as Gram-negative and Gram-positive bacteria, as well as viruses, fungi, or parasites. They also play a role in activating the immune system by modulation of signaling pathways and inflammatory response [1,2]. Moreover, they participate in tumorigenesis, due to their proliferative or suppressive properties relative to different cells [2]. In some conditions they contribute to maintaining homeostasis, while in other situations they contribute to disorders of the body’s functions and the development of different pathologies [3]. Interesting proteins with promising functions and the potential to control diseases of different etiopathogenesis (infectious or inflammatory) are defensins. Human defensins, in addition to the functions mentioned above, can also act chemotactically by interacting with C-C Chemokine Receptor Type 6 (CCR6) and recruiting immunocompetent cells with this receptor such as memory T-cells and immature dendritic cells [4]. One of them is β-defensin 2 (hBD-2) which was first described in 1997 as an antimicrobial peptide in human skin [5]. hBD-2 is a low molecular weight, cysteine-rich cationic peptide [6], known also as skin-antimicrobial peptide 1 (SAP1), encoded by the *DEFB4A* and *DEFB4B* genes, located on human chromosome 8. The structure of hBD-2 consists of 64 amino acids. hBD-2, like other proteins from the defensin family, contains in its structure a folded β-sheet, in which six cysteine residues are involved in the formation of three disulfide bonds, which in turn play a key role in maintaining the proper structural integrity of the protein [7]. hBD-2, similar to hBD-1 and hBD-3, demonstrates positive net charges in its primary structure, but differs between individual defensins. hBD-3 has the highest positive charge (+11), the next is hBD-2 with a charge of +6, and the lowest positive charge has hBD-1 (+4) [8]. This phenomenon may reflect the antimicrobial efficacy of specific peptides using electrostatic bonds to attach to negatively charged molecules present on bacterial cells, such as sialic acid [9].

Various types of epithelial cells have the ability to secrete this protein in humans, mainly as a consequence of contact with microorganisms or action of different pro-inflammatory cytokines [6], as discussed below. In hBD-2 expression, induced by pathogenic factors like bacterial LPS (lipopolysaccharide) or fungal infections, an essential role is played by protein complexes acting as transcription factors: nuclear factor kappa-light-chain-enhancer of activated B cells (NF-κB) and activator protein 1 (AP-1) [10,11]. They act by binding to the hBD-2 promoter, which is required for induction of the gene for this protein. In some tissue cells, the action of both factors appears to be synergistic, and inhibition of either pathway blocks defensin expression [12]. In contrast, other cells, such as intestinal epithelial cell lines, retain the ability to express hBD-2 despite blocking one of the pathways [13]. Recently, the role of the pathways in which the mitogen-activated protein kinases (MAPK) are involved has also been emphasized. The stimulation of Toll-like receptors (TLRs), especially TLR2 and TLR4, on specific cells by pathogen-associated molecular patterns (PAMP) is also crucial for the activation of hBD-2 expression in the course of infection [14,15].

In addition to its known antimicrobial activity and its vital role in the various epithelial tissues, hBD-2 can also be involved in chemotaxis of immune cells and activation of Toll-like receptors (TLRs) on their surface [16]. Furthermore, hBD-2 has the ability to strongly bind to the complement component C1q. Interestingly, this protein does not significantly affect the alternative activation pathway, but can inhibit the classical complement activation pathway, and may be involved in the protection against the uncontrolled activation of the innate immune response [17].

**Figure 1:**
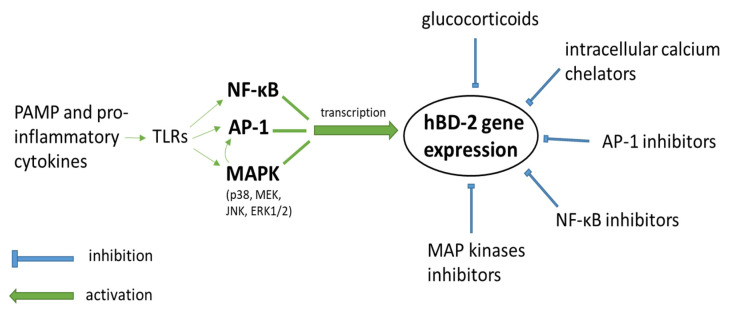
The most frequently described pathways of activation and inhibition of hBD-2 gene expression.

Given the importance of hBD-2, its properties, and the complicated mechanisms of its activation, which are presented in Figure 1, we tried to discuss in this review its ambiguous role in the human body.

## 2. hBD-2 and Oral Cavity Epithelium

The cells lining the oral cavity are continuously exposed to mechanical damage, which can easily lead to the development of various infections. Only two years after the discovery of the discussed molecule, it was shown that it is an important defense component in the oral cavity—hBD-2 is secreted by epithelial cells of the gingival mucosa, which is confirmed both by the expression of mRNA for hBD-2 in these cells and the presence of its product in saliva [18]. It has been shown that the increase of hBD-2 production in an oral mucosal culture model may be a result of an action of human milk oligosaccharides [19], which may be important in improving immunity in infants. Interestingly, studies involving children aged from 3 to 16 showed that the levels of both hBD-1 and hBD-2 in saliva increased with age, which concurs with the maturation of the immune system [20]. Gingival biopsies showed that hBD-2 is secreted at a very low level by gingival tissue in healthy people with no signs of inflammation [21]. However, production and secretion of hBD-2 by the oral keratinocytes occurs mainly after stimulation by pro-inflammatory cytokines, such as tumor necrosis factor α (TNF-α), interleukin 1β (IL-1β), and interferon γ (IFN-γ), or as a result of action of bacterial endotoxins, e.g., lipopolysaccharide (LPS), which is a significant difference from hBD-1 which is secreted continuously [18,22,23,24]. What is more, only two to four hours after contact with bacterial products, especially the *Fusobacterium nucleatum* cell wall, and with pro-inflammatory cytokines (e.g., TNF-α), the synthesis of this protein increases significantly [25]. Although NF-κB is involved in this process, it is neither sufficient or necessary for hBD-2 induction, because p38 and c-Jun N-terminal kinase (JNK) factors play a key role in the activation caused by *F. nucleatum* [26]. Additionally, in studies evaluating the effect of *Actinobacillus actinomycetemcomitans*, a bacterium involved in difficult-in-treat periodontitis, on the secretion of antimicrobial peptides, it was noticed that hBD-2 overexpression occurs through the MAP kinase pathway. More specifically, one of the bacterial outer membrane proteins (Omp100) activated this process by binding to extracellular matrix fibronectin, which is involved in the activation of these kinases. Additionally, this process was enhanced by the action of pro-inflammatory cytokines (TNF-α and IL-8) as response to infection [27]. This relationship has been noticed in the case of microorganisms inhabiting the oral cavity in the physiological state which cause enhancement of hBD-2 secretion using different pathways than pathogenic bacteria, because they use the stress- and cytokine-dependent, JNK and p38 mitogen-activated protein kinase pathway [21]. The appropriate intracellular concentration of calcium ions in gingival cells also seems to be of indispensable importance [28]. Among the stimulators of hBD-2 (*Porphyromonas gingivalis*-derived LPS, TNF-α, and combination of both), the strongest stimulating effect on the human gingival epithelial cell line was exerted by the combined action of both above-mentioned pro-inflammatory factors [23]. However, some pathogenic bacteria, such as *Neisseria meningitidis*, do not seem to stimulate the innate immune response as demonstrated by the lack of influence of bacteria incubation with pharyngeal epithelial cells on the secretion of hBD-1 and hBD-2. Additionally, the presence of *N. meningitidis* had a suppressive effect on the commensal *Lactobaccilli*-induced (*L. salivarius* and *L. reuteri*) synthesis of hBD-2. This is due to enhancement of the expression of A20 (molecule that is an inhibitor of NF-κB pathway), caused by *N. meningitidis* infection [29]. Other studies indicate that hBD-2 is effective in killing cells of *N. meningitidis*, but this is a rather ineffective process because it takes place only after two hours of incubation and does not affect the membrane. On the other hand, hBD-2 shows much lower lytic efficiency against bacteria in aggregates, which suggests defensive action of the biofilm-forming pathogen against the mechanisms of innate immunity [30]. 

Fungal infections can also modulate the components of the innate immune response in the oral cavity. Recently, studies have been published that assessed the fluctuations of inflammatory markers under the influence of *Candida* in an in vitro model of dental stomatitis, a disease often associated with the use of dental prostheses. The contact of human palate epithelial cells (HPEC) with *C. albicans* caused an increased level of hBD-2 and nitric oxide (NO) in culture supernatants as well as hBD-2 mRNA expression. However, only the levels of the described protein were maintained after a longer period of time (10 h of incubation) [31].

Studies with the use of tissue biopsy obtained from patients suffering from oral lichen planus have revealed enhancement in the hBD-2 secretion, compared to healthy controls. Importantly, it is postulated that histamine may be involved in this process because it enhances the TNF-α-mediated increase in hBD-2 mRNA expression (possible pathway through the activation of H4 receptor (H4R) on the cell surface). The role of defensin in the pathogenesis of oral lichen planus implies its possible therapeutic application [32].

Interestingly, it has been suggested that smoking may play a role in the development of periodontal diseases by significantly reducing hBD-2 synthesis, with overproduction of highly chemotactic IL-8, as demonstrated by exposure of gingival keratinocytes to cigarette smoke [23]. However, due to one of the methods of treating periodontal lesions in the oral cavity, namely low-dose laser therapy, it is possible to increase the secretion of hBD-2 by the oral epithelial cells in the transforming growth factor (TGF)-β1–related pathway. The mentioned factor acted mainly on oral fibroblasts, and to a lesser extent on human oral keratinocytes [33].

## 3. hBD-2 and Gastric Mucosa

One of the most common and challenging conditions is stomach infection with *Helicobacter pylori*. This bacterium colonizes the gastric epithelium and contributes to the development of cancer of this organ, as well as gastric and duodenal ulcers. Furthermore, it may weaken the immune system of the host, allowing it to survive in the body throughout life [34,35,36,37,38]. The epithelium of the stomach, like other parts of the digestive system, has the ability to synthesize and secrete a wide variety of antimicrobial peptides, including defensins [39]. hBD-2 and hBD-3 are expressed in enterocytes ‘on demand’ [40]. In vitro studies showed that cytotoxic *H. pylori* caused a marked increase of hBD-2 expression by two gastric epithelial cell lines (MKN7 and AGS; both are human gastric adenocarcinoma cells), whereas hBD-1 expression showed a much weaker increase [41]. Overexpression of hBD-2 induced by *H. pylori* has also been demonstrated in gastric tissue biopsy [42]. Increased production of this protein in *H. pylori* infection was also confirmed by a significant increased concentration of hBD-2 in the gastric juice of infected patients, compared to the control group of healthy people. Moreover, a positive correlation between these concentrations and the degree of inflammatory infiltration by neutrophils and mononuclear cells in the corpus of stomach in histological examination was found [43]. Additionally, ex vivo studies confirmed a significant upregulation of hBD-2 expression in the gastric mucosa of infected patients, while the level of secreted hBD-3 was significantly lowered [44]. This suggests various modulating properties of this bacterium. In vitro studies with *H. pylori* cell suspension showed the antibacterial activity of synthetic hBD-2 at various concentrations, and in the best case (hBD-2 concentration 10^−5^ M), bacteria were completely eradicated [42]. However, despite the increase of human β-defensin 2 synthesis during infection, bacterial eradication has not been confirmed in patients [45]. While this peptide, along with other components of mucosal and innate immunity, like low pH of gastric juice, protect the epithelium of the stomach and gastrointestinal tract, *H. pylori* has the ability to adapt to this condition, colonize the gastric mucosa and induce inflammation [45,46]. This peptide accumulates on the bacterial cell surface, and despite small structural changes and minimal growth inhibition, it does not eradicate them effectively, while such resistance may result from structural modifications of the cell membrane [45,47]. On the other hand, there are suggestions for the possible elimination of other pathogenic microorganisms susceptible to the effect of this protein during this infection [45], which may prevent superinfections in an already weakened host organism. Meanwhile, it was suggested that up-regulation of hBD-2 in the situations described above can be used as a marker of inflammation of the gastric mucosa [48].

## 4. hBD-2 and Intestinal Epithelium

Intestinal epithelial cells, together with secreted antimicrobial peptides, constitute an effective barrier against the entry of pathogens into the host organism [49,50]. Maintaining the balance between attacking pathogenic bacteria and conserving and supporting commensal microbiota is crucial in maintaining homeostasis. Some reports describe the role of β-defensins secreted by the colonic epithelium in protecting against not only bacterial, but also fungal, parasitic, and viral infections [51]. Recently, the in vitro enhancing effect of hBD-2 on the integrity of the intestinal epithelium has been described, e.g., by improving tight junctions between cells. This made it possible to increase protection against the invasion of pathogens such as *C. albicans* from the intestinal lumen to other parts of the body [52]. In vitro studies have shown (similar to other epithelial cells) that cells from the colon epithelial lines, human colon adenocarcinoma cells (Caco-2) and human colorectal adenocarcinoma cells (HT-29), consistently secrete small amounts of the hBD-1 protein, which has also been confirmed by mRNA expression studies [53]. In contrast, hBD-2 mRNA expression and protein production by the intestinal epithelium is significantly increased after stimulation with enteroinvasive bacteria, such as *Escherichia coli* O29:NM serotype and *Salmonella dublin*, as well as *Bacteroides fragilis* enterotoxin and pro-inflammatory cytokine IL-1α, in a NF-κB pathway dependent manner, while it is imperceptible in unstimulated cells [13,53]. What is more, in the *B. fragilis*-dependent activation of hBD-2 expression, the presence of AP-1 was irrelevant because the induction of the hBD-2 gene was associated with the aforementioned NF-κB, as well as with activation of MAP kinases due to the phosphorylation of extracellular signal-regulated kinase ½ (ERK1/2), JNK and especially p38 [13]. Moreover, in the activation of ERK-associated hBD-2 expression in intestinal epithelial cells in response to, e.g., IL-1α, isoleucine appears to be of vital importance. However, this amino acid incubated with cells in the absence of pro-inflammatory cytokines does not activate this pathway [54]. Other studies with the use of Caco-2 cells have well illustrated the selective action of antimicrobial peptides, hBD-2 and hBD-3, against pathogenic strains of *Salmonella typhimurium*, without changing the number of colonies of commensal *Enterococcus faecium* strain, and even increasing its protective effect as a probiotic [55]. It appears that in the intestinal epithelium the hBD-2 promoter is activated in a TLR2-dependent manner when it is stimulated by bacterial peptidoglycan, or a TLR4-dependent manner by LPS stimulation. This may be an explanation for the fact that the human colon adenocarcinoma SW480 cells that express TLR may respond better to LPS or peptidoglycan stimulation than Caco-2 and T84, which weakly express these receptors [15].

Interesting data provide evidence for the effect of probiotic bacteria, *Lactobacillus rhamnosus* and *Bifidobacterium longum*, on increased hBD-2 secretion and decreased IL-8 production on the protection of intestinal cells against *P. aeruginosa* infection [56]. However, prolonged infection of *P. aeruginosa* PAO1 strain also suppressed IL-8 secretion and increased hBD-2 secretion in intestinal epithelial cells, following increased mRNA expression for both molecules. The involvement of NOD1 (nucleotide-binding oligomerization domain 1) in overexpression of hBD-2 after infection with this pathogen has been demonstrated [57].

Parasitic infections also interfere with innate immunity. Infection with the intestinal parasite *Cryptosporidium parvum* can affect defensins in a different manner. In the course of infection, it results in the attenuation of the secretion of murine beta-defensin-1 (mBD-1) in an animal model of infection, which was confirmed by a reduction in the expression of constitutively secreted hBD-1 in in vitro studies using the HT-29 cell line. In contrast, mBD-3, which is the equivalent of human BD-2, was secreted in the same high level in mice regardless of infection, while HT-29 cells, which do not express it under normal conditions, show high expression following infection [58]. Such interference with hBD-1 levels may enable the parasite to escape the host’s immune defense, while hBD-2 remains ‘in fight’ and its elevated levels indicate a stimulation of part of the immune system and perspectives to effectively eliminate the cause of infection, especially as the effect of AMPs, including hBD-2, in efficiently inactivating *C. parvum* sporozoites has been reported [59]. Another parasite associated with intestinal infection, causing damage of the intestinal mucosa, severe diarrhea, and, as a consequence, parental infections, i.e., *Entamoeba histolytica*, can also interfere with the host’s immune system. In vitro exposure of Caco-2 cells to this pathogen results in both increased mRNA expression and hBD-2 protein secretion, which was caused by activation of TLR2 and TLR4 on the surface of intestinal cells and with the stimulation of the NF-κB pathway [60].

Inflammation of the intestines caused by various etiological factors is concurrent with the disturbance of both the gut microbiota and the level of antimicrobial peptides [61]. Many studies also suggest the role of human β-defensin 2 in the pathogenesis of inflammatory bowel disease (IBD). The main representatives of this group of diseases are Crohn’s disease (CD) and ulcerative colitis (UC). Studies with the use of both the Caco-2 cell line and colon biopsy tissue from IBD patients indicate constitutive production of hBD-1 independent of inflammation [62]. On the other hand, a clear increase in hBD-2 mRNA expression by Caco-2 cells was observed after stimulation with pro-inflammatory cytokines [62], which has been repeatedly confirmed in biopsies of patients with inflammation, especially in UC, compared to the control groups of healthy individuals [62,63,64]. The important cells producing and releasing defensins, such as hBD-2, hBD-3, and hBD-4, are mature plasma cells residing in the lamina propria of the colon, the number of which is several times higher in UC patients than in CD or healthy patients [65]. Other studies indicate elevated levels of hBD-2 in the sera obtained by patients suffering from Crohn’s disease, which may be caused by the overproduction of inflammatory mediators (TNF-α, IFN-γ, and IL-22) as a result of additional parental inflammations common in this disease [66]. However, in the same studies, no increased secretion of the hBD-2 peptide in terminal ileum biopsies in patients with CD was demonstrated, while the level of hBD-3 was increased four-fold, but without significant changes in mRNA expression [66]. Interestingly, it appears that patients with irritable bowel syndrome (IBS) without micro- and macroscopic signs of inflammation also have elevated fecal hBD-2 values, comparable to those in UC patients [67].

In recent years, several murine models of experimentally induced enterocolitis have been described, which demonstrate the efficacy of β-defensin 2 as a therapeutic agent in alleviating disease symptoms. Subcutaneous administration of hBD-2 to mice with colitis caused by supplementing the water with dextran sodium sulfate (DSS), colitis induced by intracolonic administration of trinitrobenzene sulfonic acid (TNBS), as well as colitis induced by transplantation of CD4+/CD25- T cells to mice with severe combined immunodeficiency (SCID), resulted in a reduction of disease markers, inter alia, by suppression of inflammation in the intestines and reducing weight loss in the animals [68]. In addition, in ex vivo studies using human peripheral blood mononuclear cells (PBMCs) stimulated with LPS or Pam3CSK4 (Toll-like receptor 1/2 agonist, inducer of the production of inflammatory factors), the anti-inflammatory effect of hBD-2 was emphasized, due to a significant reduction in the secretion of pro-inflammatory cytokines, such as TNF-α, IL-1β, IFN-γ, IL-23, and IL-12p70. The suppression of inflammation was also achieved due to the interaction of hBD-2 with CCL2 receptors on dendritic cells, which resulted in the weakening of NF-κB activity and an increase in cAMP response element-binding protein (CREB) through its phosphorylation [68]. The same animal models have also been used previously to demonstrate the therapeutic effect of recombinant hBD-2 produced using *Saccharomyces cerevisiae* and administered subcutaneously. The main indicators of improvement in the condition of the mice were a significant reduction in tissue damage in the histological examination, a decrease in weight loss and improvement in other parts of the disease activity index, i.e., stool consistency and hematochezia. As in the study described above, the effect of defensin on LPS-stimulated human PBMCs was marked by a reduction in synthesis of pro-inflammatory cytokines (TNF-α, IL-1β, and IL-23) as well as increasing the release of anti-inflammatory cytokines (IL-10 and IL-24) [69]. Furthermore, orally administered hBD-2 showed a therapeutic effect in a murine model of DSS-induced colitis, and modulation in the composition of the gut microbiome was also reported [70], which is most often disturbed in the course of IBD [71,72,73]. These results suggest the influence of therapeutic administration of hBD-2 not only on recovery, but also on the balance of systemic homeostasis (based on the maintenance of an appropriate composition on the microbiome).

Interestingly, very early pre-clinical in vitro studies using the Caco-2 cell line indicate a possible immunomodulatory effect of T4 bacteriophage, evidenced as a marked increase of hBD-2 gene (*DEFB4A*) expression [74]. These results may suggest the possible use of phages as modulators of the immune response in assisting the treatment of diseases such as IBD via induction of hBD-2.

## 5. hBD-2 and Cells from Central Nervous System

For a long time, attention has been paid to the communication of the immune system with the nervous system [75,76,77]. A significant role of T-cells, B-cells, cytokines or various type of receptors in the development of the central nervous system has been described [78]. Of the different types of nerve cells in the brain, only astrocytes are susceptible to synthesis of hBD-2 mRNA and release of the hBD-2 protein in vitro under the influence of LPS and cytokines (TNF-α and IL-1β). However, almost all investigated cell cultures (astrocytes, meningeal fibroblasts and microglia, excluding neurons) were able to constitutively express hBD-1 mRNA under normal conditions [79]. In brain capillary endothelial cells (BB19 cell line), the inducing effect of *Chlamydophila pneumoniae* infection in the increase of hBD-2 secretion (increase in both mRNA expression and peptide production) has been reported [80]. This suggests that defensins may also play an important role in immune defense in cerebral blood vessels.

It has been suggested that disturbances in antimicrobial peptide levels, including hBD-2, may be important in the development of pathological conditions in the central nervous system, which include disturbance of cell maturation (such as dendritic cells) or overexpression of pro-inflammatory cytokines [81].

## 6. hBD-2 and the Epithelium Lining the Respiratory System

Not without significance is hBD-2 expression in epithelial cells lining the lung and serous cells of the lung submucosa glands [82]. It has been proven that the LPS-induced activation of hBD-2 gene expression in lung cells requires NF-κB and AP-1 protein complexes, and also intracellular calcium ions are essential in this process [10]. A multiple and wide influence of human β-defensin 2 is postulated in various diseases of the respiratory tract [83]. Recent studies indicate significant associations in the mutations of hBD-2 encoding genes (*DEFB4A* and *DEFB4B*) with the development of atopic asthma in children [84]. Moreover, the additional association of hBD-2 with asthma is due to the fact that glucocorticoids (such as dexamethasone), drugs commonly used in the treatment of asthma [85], can attenuate the expression of the human β-defensin 2 gene in human airway cells [10] (however there are different data from studies with the use of Caco-2 cells, in which dexamethasone enhances the hBD-2 mRNA level [86]). Interestingly, the protective effect of orally administered hBD-2 has been reported in the course of atopic asthma and house dust mite allergy in a mouse model [84]. Serum studies of healthy individuals, as well as patients with chronic *Pseudomonas aeruginosa* infection of the lower respiratory tract and suffering from idiopathic pulmonary fibrosis, showed that in patients with infection, especially those caused by mucosal strains, the levels of hBD-2 and hBD-3 are most clearly marked when compared to other groups [87]. It has been suggested that the hBD-2 level in plasma may be a useful predictor in the assessment of an unfavorable course of pneumonia [88]. In contrast, significant amounts of hBD-2 and hBD-4 were detected in the epithelial lining fluid of patients with mucoid *P. aeruginosa* infection, while they were not detectable in other groups. What is more, these concentrations correlated with serum C-reactive protein (CRP) levels [87]. In fact, the presence of TLR4 receptors on lung epithelial cells plays a critical role in bacterial LPS-stimulated expression of hBD-2 by these cells [89]. The respiratory epithelium along with secreted antimicrobial peptides may also play an important protective role against life-threatening fungal infections of the lungs. It appears that exposure of human type II alveolar epithelial (A549) and human bronchial epithelial (16HBE) cells to *Aspergillus fumigatus*, especially swollen conidia, increases expression of the hBD-2 and hBD-9 genes, as well as the level of hBD-2 in culture supernatants [90]. 

In vitro studies using human lung epithelial cell lines show interesting results regarding the influence of *Brucella abortus* infection on antimicrobial peptides (AMP) secretion. While A549 and Calu-6 (bronchial epithelial cell line) cells expressed CCL2 in response to infection, the increase of hBD-2 secretion by A549 cells occurs upon incubation with monocytes infected with this bacterium. In fact, the release of hBD-2 was related to the secretion of IL-1β by infected monocytes. Nevertheless, cytokine-dependent hBD-2 secretion does not result in effective antibacterial activity against *B. abortus* strains [91], therefore, in this case, it is only possible for this peptide to interact in an immunological pathway without effectively contributing to the killing of this pathogen.

Different levels of hBD-2 secreted by lung tissues may occur in smokers and non-smokers. In a rat model of exposure to cigarette smoke, increased expression of rat beta-defensin-2 (rBD-2) mRNA and clearly increased levels of this protein in lung tissue have been reported [92]. This suggests an important role for hBD-2 in the protection of the respiratory tract against oxidative stress, as well as related infections and development of chronic obstructive pulmonary disease, which are a consequence of exposure to cigarette smoke. 

Some studies also highlight the antiviral effect of hBD-2 [93]. Recent *in silico* studies indicate the ability of human beta-defensin-2 to bind the receptor binding motif (RBD) domain, that complexes with angiotensin-converting enzyme 2 (ACE2) and mediates SARS-CoV-2 entry into host cells. Due to this interaction of the small peptide like hBD-2, it is possible to block the entry of the virus [94]. However, the antiviral activity of beta-defensins (hBD-2 and also hBD-5 and hBD-6) after prior viral entry into cells has not been proven [95]. These results indicate a possible protective effect of hBD-2 against viral infections in the lungs even before pathogen penetration. 

Furthermore, hBD-2 has been suggested to have role in controlling SARS-CoV-2 infection [96]. In fact, patients with COVID-19 have markedly reduced serum levels of hBD-2 which can promote virus persistence and disease severity [97].

## 7. hBD-2 in Skin and Wounds

The largest organ in the human body and simultaneously the most important barrier protecting against entry of pathogens is the skin. Among the wide range of very important functions of the skin is production and secretion of various antimicrobial peptides. As was mentioned above, hBD-2 is first described in the skin. The role of hBD-2 in various skin infections and non-infected diseases was also highlighted. Studies on the primary keratinocytes revealed that in the IL-1β- and *P. aeruginosa*-induced activation of hBD-2 gene expression, the synergistic action of the NF-κB and AP-1 pathways is crucial [12]. There are studies which suggest that hBD-2 may be a marker of severity of atopic dermatitis due to their pronounced correlation with disturbances in stratum corneum and lesional skin [98]. Moreover, levels of hBD-2 per µg of total protein in the sample was the highest in lesion skin from atopic patients, compared to levels in patients without lesions and healthy people [99]. In contrast, other studies show no differences in the levels of various AMP, including hBD-2 and human cathelicidin LL-37 in atopic patients compared to healthy individuals [100]. Due to the fact that patients suffering from atopic dermatitis are very exposed to various skin infections [101], the levels of defensins secreted by them may be an important factor influencing the course of disease and possible prevention of infection.

Another chronic relapsing inflammatory disease of the skin is *psoriasis vulgaris*, in which keratinocytes, T cells, and dendritic cells participate [102], and which is considered to be an autoimmune disease [103]. Many beta-defensins (hBD-2, hBD-3, hBD-4, and also LL-37, excluded hBD-1) have been shown to contribute to the increased secretion of IL-18 and expression of its mRNA by primary human keratinocytes [104], and this interleukin is important in the development of skin diseases [105]. Therefore, apart from the positive effects of HBD-2, their role in exacerbating the symptoms of the disease is also emphasized [106]. Activation of keratinocytes to the overexpression of IL-18 takes place via the p38 and ERK1/2 MAPK dependent pathway, without the participation of JNK in this process [104]. It turns out that the beta-defensin gene copy number is highly correlated with the risk of developing psoriasis [107]. Interestingly, plasma hBD-2 levels may also play a role in cutaneous diseases. It has been shown that in patients suffering from psoriasis, serum levels of the hBD-2 protein strongly correlated with the degree of disease activity (based on the psoriasis area and severity index—PASI), which was not observed in patients with atopic inflammation in the same study [108]. It has been suggested that measuring the concentration of this protein in serum may be an indicator of the severity of psoriasis [108] and effectiveness of treatment with Janus kinase (JAK) inhibitors [109]. Interestingly, a correlation between the increased amount of neutrophil extracellular traps (NETs) in cutaneous psoriasis lesions and the increased amount of hBD-2 therein was described. Moreover, stimulation of normal human keratinocytes with netting neutrophils from psoriasis patients resulted in enhanced synthesis of both mRNA and hBD-2 protein, and such an induction mechanism of one of the AMPs may to some extent explain the enhanced antimicrobial protection of psoriatic plaques [110].

Promising data suggest the possibility of using recombinant beta-defensins in wound healing. The results point to hBD-2 with the appropriate substitutions (A-hBD-2) showing greater stability, lower cytotoxicity and better antimicrobial activity than hBD-2 in in vitro keratinocyte cell line studies. Furthermore, the migration and proliferation of these cells was promoted, and many cytokines were secreted through the activation of phospholipase C. The A-hBD-2 wound healing effect was confirmed in an in vivo rat model [111]. Other studies have illustrated the effect of 1,25-dihydroxyvitamin D3 on an increase in mRNA expression and protein secretion of both hBD-2 and LL-37 in primary diabetic foot cell cultures, in which AMP levels are typically decreased. The antimicrobial activity of the culture supernatants and their influence on the migration of keratinocytes confirmed the possible usefulness of such a treatment strategy in the wound healing process [112]. Examples of diseases in which the action of hBD-2 may result in positive effects, and disorders in which hBD-2 may play a role of diagnostic marker are presented in Table 1 and Table 2, respectively.

Evidently, in skin cancers accompanied by significant changes in the composition of the bacterial communities present on the skin, the microbiome may be associated with the proliferation of tumor cells. In biopsies from patients with a keratinocyte skin tumor, overabundance of *Staphylococcus aureus* was associated with overexpression of hBD-2 and, importantly, with increased tumor proliferation [116]. It can be presumed that the overproduction of defensins has an indirect influence on the progression of neoplastic processes.

## 8. hBD-2 in Pregnancy and Infancy

Beta-defensins, along with other components such as mucins, neutrophil gelatinase-associated lipocalin (NGAL) or antibodies (IgG and IgA), constitute a relevant part of the antimicrobial barrier of the vaginal environment [117]. In pregnant women, expression of the human β-defensin 2 gene positively correlates with the concentration of this protein in cervical samples [118]. Moreover, in the example for *E. coli* strains, the influence of hBD-2 level on the antimicrobial activity of cervicovaginal secretion was described [118], which indicates the role of this protein in maintaining the proper immune state of the vaginal mucosa during pregnancy. A similar enhancement in the secretion of hBD-2 has been demonstrated in the infection with this bacterium [119] as well as with *C. albicans* [120] of the extra-placental membranes—the amniotic and choroid, especially in the choriodecidual region. Women in early pregnancy who suffer from bacterial vaginosis have significantly lower hBD-2 concentrations in vaginal samples compared to healthy women [113]. In contrast, increased secretion of hBD-2 by the human vaginal epithelial cell line (VK2/E6E7) after stimulation with *Candida albicans* was described in in vitro studies [121]. In studies of the amniotic fluid, hBD-2 was present in all samples, but its concentration increased significantly during intra-amniotic infections [114]. In addition, hBD-2 mRNA expression was detected in placental, chorion and villus tissues [122]. In summary, the appropriate level of antimicrobial peptides, in particular hBD-2, in the female reproductive system, can protect pregnant women against premature rupture of membranes (PROM) and preterm delivery [113], and it is also an important element of innate immunity.

What is more, levels of beta-defensins are very important in postmenopausal patients in some disorders, like lichen sclerosus, because decreased concentration of hBD-1 and significantly increased levels of hBD-2 and hBD-3 in these women are described. Overproduction of defensins translate into increased activation of fibroblasts and synthesis of the extracellular matrix, which promotes disease progression. This suggests the possibility of treating such conditions by modulating the composition of the microbiome contributing to this condition [115].

The beneficial effects of breastfeeding on the development of newborns’ immune system and protection against various infections both during and for a long time after cessation of feeding are discontinued known [123,124,125]. The significant role of human β-defensin 2 in these processes has also been emphasized. It has been shown that colostrum contains higher levels of hBD-2 than mature milk [126,127,128]. Additionally, it turns out that the method of delivery was important for the content of this protein in breast milk, which is manifested by significantly higher levels of hBD-2 in the case of vaginal delivery, than in women undergoing cesarean section [126]. Significant amounts of antimicrobial peptides, such as lactoferrin, hBD-1, and hBD-2, are also present in preterm breast milk, with clearly higher concentrations determined on day 7 than on day 21 after delivery [129]. The importance of the presence of hBD-2 in breast milk for the antimicrobial protection of newborns is confirmed by in vitro tests, which showed that this milk-derived protein has clear bactericidal properties against various dangerous pathogens, like *E. coli*, *Salmonella* spp., *P. aeruginosa*, *Serratia marcescens*, or *Acinetobacter baumannii* [128]. On the other hand, the levels of hBD-2 in breast milk are not related to the development of allergies in children from high-risk groups [127].

In addition to maternal protection, the production of hBD-2 by various tissues of newborns is of great importance for their homeostasis and for maintenance of health. Preterm neonates have lower levels of hBD-2 in serum than term newborns, and this translates into a proven higher risk of developing life-threatening sepsis [130]. However, hBD-2 concentrations in bronchoalveolar lavage fluid collected from premature and mature infants did not differ in relation to the gestational age, but production of this protein was up-regulated in pneumonia and systemic infections [131].

## 9. hBD-2 and Genitourinary Tract

Research has shown that hBD-2 is produced in distal nephron epithelial cells and can be detected in urinary tract infections [132]. Caterino et al. (2015) conducted a study on a group of 40 patients with an average age of 57 [133]. In addition, comorbidities such as heart disease, diabetes, and stroke were reported in patients. Furthermore, there were also people on immunosuppression, with cancer or organ transplants. 75% of patients complained of problems with the urinary tract. Urine tests were performed among the patients, which revealed the presence of bacteria (*Citrobacter* spp., *S. aureus*, *E. coli*, *Klebsiella pneumoniae*, *Lactobacillus*-like, *Staphylococcus saprophyticius*, *Morganella morganii*, *Gardnerella vaginalis*-like, *Enterococcus faecalis*) in 13 of them. hBD-2 levels were tested in 35 people and found to be significantly higher in people with positive urine cultures than in people without infection. Lehmann et al. (2002) examined the expression of hBD-2 genes in samples of renal tissue from nephrectomy [134]. They were obtained from 15 patients with a mean age of 60 years with chronic infections of the upper urinary tract. Bacteria such as *E. coli*, *Klebsiella* and *P. aeruginosa* were isolated from the urine of the patients. The performed studies showed that the genes encoding hBD-2 were expressed in 14 out of 15 samples. Staining of samples with hBD-2 antiserum showed the presence of the hBD-2 protein in distal tubules, loops of Henle and collecting ducts throughout the cortex and medulla. In addition, incubation of human kidney carcinoma cell line (CAL-54) cells with 10 ng/mL IL-1β for 6 hours resulted in a 476× increase in hBD-2 mRNA expression compared to unstimulated cells. Incubation of cells with 10 ng/mL TNF-α resulted in an 82× increase in hBD-2 mRNA transcription levels, while the addition of 100 µg/mL *E. coli* LPS led to a 15× increase in hBD-2 mRNA transcription levels compared to negative controls. As shown in the above studies, hBD-2 is expressed by urinary tract epithelial cells as a result of contact of the host cells with microorganisms or pro-inflammatory cytokines [6].

Human β-defensin 2 also plays an important role in the immune defense of the prostate tissues. It has been shown that hBD-2 mRNA is constitutively expressed by cells from the human prostate epithelial (RWPE-1) and human prostate cancer (DU-145) cell lines, and also by tissue obtained from patients with benign prostatic hyperplasia. After LPS stimulation, hBD-2 was upregulated, and this process involved NF-κB factor activated by phosphorylation [135]. Moreover, LPS-induced overexpression of hBD-2 is mediated by zinc ions, which are associated with phosphorylation of ERK1/2 and p38 factors and increasing their total form. This provides evidence for the participation of not only the mentioned NF-κB but also signaling by MAP kinases in the induction of defensin following bacterial infection in the tissues of the prostate [136].

The individual factors causing changes in human β-defensin 2 levels in different tissues along with the directions of these changes are presented in Table 3.

## 10. Conclusions

Recent progress in studies on hBD-2 suggest that this agent may play an important role in the pathology of human diseases. Experimental and clinical observations suggest that in some clinical settings hBD-2 may be a marker of exacerbation of disease, which can also suggest induction of biological defenses against an invading pathogen. Moreover, the data presented in this review indicate that hBD-2 appears to be a very promising agent with significant therapeutic potential in a variety of human disorders. Preliminary data suggesting that phages may enhance its production by epithelial cells are interesting and, if confirmed, may shed new light on phage interactions with eukaryotic cells and their implications. Further studies are warranted to establish the place of hBD-2 in pathophysiology and its therapeutic potential.

## Figures and Tables

**Table 1:** Diseases in which hBD-2 may induce beneficial effects.

Conditions Where Beneficial Effect of hBD-2 Is Suggested and Proposed as a Therapeutic Agent	
fungal intestinal infection [52]	upregulation of intestinal epithelium integrity
experimental colitis [68,69,70]	suppression of inflammation
premature delivery [113]	protective effect
atopic asthma [84]	protective effect
house dust mite allergy [84]	protective effect
smoking-induced cell damage [23,92]	protection of the respiratory tract against oxidative stress and infections
wound healing [33,111,112]	increasing the migration and proliferation of cells
oral lichen planus [32]	enhancement of the level of hBD-2 reduced by the disease
virus diseases [94,95]	possible inhibition of virus entry into the host cells

**Table 2:** Diseases in which hBD-2 may be a diagnostic marker.

Conditions Where Elevated Levels of hBD-2 were Noted and Proposed to Be a Marker
gingival inflammation [18,22,23,24,25]
infection with *H. pylori* [41,42,43,44,45,46]
ulcerative colitis [62,63,64]
Crohn’s disease [66]
irritable bowel syndrome [67]
intra-amniotic infections [114]
lichen sclerosus [115]
smoking [92]
atopic dermatitis [98,99]
psoriasis [107,108]

**Table 3:** hBD-2 gene expression and protein levels in different tissues in relation to external factors and mediators.

Tissue/Cells	Stimulus	mRNA hBD-2	hBD-2 Protein
oral keratinocytes/oral epithelial cells	TNF-α IL-1β IFN-γ LPS IL-2, IL-6 *C. albicans*	↑ * [18,22,23,25,31]/0 ** [29]	↑ * [18,22,23,25,31]
	human milk oligosaccharides	-	↑ [19]
	cigarette smoke	↓ [23]	↓ [23]
	laser therapy	↑ [33]	↑ [33]
gastric epithelial cell	*H. pylori*	↑ [41,42,44,45]	↑ [43]
colon epithelial cells	IL-1α IL-22 bacteria enterotoxins parasites	↑ [13,53,60,62]	↑ [13,53,60,62]
	probiotic bacteria	↑ [56]	↑ [56]
	T4 bacteriophage	↑ [74]	-
genitourinary tract	fungi bacteria LPS IL-1β TNF-α	↑ [118,134,135]	↑ [113,118,133,135]
placental tissue	fungi bacteria	-	↑ [119,120]
astrocytes	TNF-α IL-1β LPS	↑ [79]	↑ [79]
brain capillary	*Chlamydophila pneumoniae*	↑ [80]	↑ [80]
lung epithelium	LPS bacterial (e.g., *Pseudomonas*) infection fungal infection IL-1β	↑ [89,90]	↑ [87,89,90]
	glucocorticoids (dexamethasone)	↓ [10]/↑ [86]	-
	*B. abortus* infections	0 ** [91]	0 ** [91]
skin keratinocytes	bacteria IL-1α IL-1β	↑ [116]	↑ [137]
	D3 vitamin	↑ [112]	↑ [112]
	NET	↑ [110]	↑ [110]

* prevailing effect; ** no alterations; ↑ stimulation; ↓ inhibition; - no data available.

## Data Availability

Not applicable.
